# Microvascular free-flap transfer for head and neck reconstruction in elderly patients

**DOI:** 10.1186/1471-2482-13-S2-S27

**Published:** 2013-10-08

**Authors:** Francesco Turrà, Simone La Padula, Sergio Razzano, Paola Bonavolontà, Gisella Nele, Sergio Marlino, Luigi Canta, Pasquale Graziano, Giovanni Dell'Aversana Orabona, Fabrizio Schonauer

**Affiliations:** 1 Unit of Plastic, Reconstructive and Aesthetic Surgery, Federico II University, Via S.Pansini 5, 80131, Naples, Italy; 2 Department of Maxillofacial Surgery, Federico II University, Via S.Pansini 5, 80131, Naples, Italy

## Abstract

**Background:**

With the increase in life expectancy, the incidence of head and neck cancer has grown in the elderly population. Free tissue transfer has become the first choice, among all the reconstructive techniques, in these cases. The safety and success of micro vascular transfer have been well documented in the general population, but its positive results achieved in elderly patients have received less attention.

**Methods:**

We retrospectively studied 28 patients over the age of 60 years. The aim of this paper was to study the success rate of free tissue transfer and investigate the complication incidence in this patient population.

**Results:**

Twenty-eight free flaps were performed to reconstruct medium to large cervico-facial surgical defects in six years. No difference was noted between success and complication rates observed between general and elderly population.

**Conclusion:**

This study indicates that free-flap technique for head and neck reconstruction could be considered a safe option in elderly patients when a good pre-operative general status is present.

## Background

Microsurgical free-tissue transfer has gained a central role in plastic surgery for difficult reconstruction of head and neck defects, modifying the treatment of cancer in this region.

Although this technique has become a safe choice, complications may occur in 5-25%; these patients may require a surgical re-exploration of the free flap [1].

The proportion of elderly people with head and neck cancer is rising due to an overall increase in life expectancy.

The safety and success of free flap transfer have been well documented in the general population; positive results achieved in elderly patients have received less attention [2].

The aim of the present study is to investigate the effect of age on the outcome of such procedures, the medical impact of prolonged surgery and if it is worthy against the functional benefits and the better life expectancy achieved by the use of microvascular reconstruction.

## Methods

We retrospectively reviewed our experience with microsurgical free-tissue transfer in patients over the age of 60 whom we arbitrarily defined as "elderly".

Between January 2007 and February 2013, 28 patients with head and neck cancer were treated at the Maxillofacial Surgical Unit and reconstructed by our Plastic Surgery team.

The patients were classified into two groups according to age: between 60 and 69 years (age group I) and between 70 and 79 years (age group II), respectively. Table 1.

**Table 1:** Patient series

Name	Age	Group	ASA	Type of tumour	Site	Reconstruction	Days in Intens.Care	Complications
PO	60	I	1	SCC	Cheek	Radial forearm	1	-
AR	60	I	2	Sarcoma	Mandible	Fibula osteocutaneous	1	Venous throbosis
GE	61	I	1	SCC	Mandible	Fibula osteocutaneous	2	-
AG	62	I	1	SCC	Palate	Radial forearm	1	-
MD	62	I	2	SCC	Tongue	Radial forearm	1	-
PM	63	I	2	SCC	Larynx and pharynx	Radial forearm	2	-
TD	64	I	1	BCC	Cheek (ext.)	Radial forearm	1	-
CG	65	I	2	SCC	Scalp	Latissimus dorsi	1	-
FS	65	I	1	SCC	Cheek (ext.)	Latissimus dorsi	1	-
DA	66	I	2	SCC	Tongue	Ulnar forearm	1	-
AP	66	I	2	SCC	Tongue	Ulnar forearm	1	-
DR	66	I	1	SCC	Floor and tongue	Radial forearm	1	-
DN	67	I	1	SCC	Floor and tongue	Radial forearm	1	-
SA	68	I	2	SCC	Cheek	Radial forearm	1	-
IB	69	I	1	SCC	Pharynx	Radial forearm	2	-
BM	70	II	2	SCC	Floor	Radial forearm	1	-
DC	70	II	1	SCC	Mandible	Fibula osteocutaneous	2	-
RA	71	II	1	SCC	Lips and cheek	Radial forearm	1	-
PA	72	II	3	SCC	Tongue	Radial forearm	3	Pulmonary failure
RM	72	II	2	SCC	Floor and tongue	Radial forearm	1	-
CR	72	II	1	SCC	Scalp	Latissimus dorsi	2	-
SG	73	II	2	SCC	Cheek	Radial forearm	1	Haematoma
SM	75	II	1	SCC	Tongue	Radial forearm	1	-
FS	75	II	2	SCC	Mandible	Fibula osteocutaneous	2	Partial necrosis
DM	75	II	1	SCC	Floor and tongue	Radial forearm	1	-
PI	76	II	2	SCC	Half right face	Rectum abodminis muscle	3	Venous thrombosis
GG	77	II	2	SCC	Half right face	Rectum abodminis muscle	2	-
TC	77	II	1	SCC	Floor and tongue	Radial forearm	1	-

The oral cavity was the most frequent site of reconstruction in all groups. All patients undergoing microsurgical free-tissue transfer were recovered in intensive care unit until their stabilization. The flap was monitored by checking paddle skin colour, bleeding and, if necessary, Doppler signal, every 2 hours for the first day, every 6h on day 2-3 and then less frequently until patient's discharge.

Many variables were analysed for each group. Our records were reviewed searching for diagnosis, free-flap type, defect site, patient age and sex, preoperative medical problem, length of operation, complications and operative mortality. We classified complications into two main clusters: technique-related (seroma, haematoma, infection, dehiscence, thrombosis, congestion and skin or flap loss) and general conditions-related. Technique complications were classified as major, requiring surgical re-exploration, or minor, not requiring re-exploration.

Long-term functional outcomes (speech, swallowing and chewing) were assessed 6 month after surgery.

## Results and discussion

A total of 28 patients (20 male, 8 female; ranging 60 to 77 years) underwent a free-tissue transfer for head and neck tumours. Fifteen patients, were aged between 60 and 69 years (age group I) and thirteen patients were aged between 70 and 79 years (age group II).

Most frequent histological diagnosis, preoperatively indicated by biopsy, was squamous cell carcinoma.

Various free flap types were used to reconstruct a variety of defects. Microvascular free flaps used were: radial forearm (n = 17), fibula (n = 4), latissimus dorsi (n = 3), ulnar forearm (n = 2), rectus abdominis (n = 2). Total success rate was 93% (26/28).

Preoperative medical problems were evaluated through American Society of Anesthesiologists (ASA) score; just one patient classified as ASA III class underwent microvascular technique.

Donor site major complications were not observed.

Total complication rate was 17,9% (5/28); complications were divided into two different groups: technique-related and systemic condition-related. Four technique-related complications were observed (14,3%): of these three were major and one minor. Major flap complications (10,7%) consisted of one venous thrombosis of the pedicle and one partial necrosis in age group II (15,4%) and one venous thrombosis in age group I (6,7%). Flap salvage was possible in the younger patient with venous thrombosis by exploring the flap and performing a new anastomosis; in the older patient with partial necrosis another local flap was needed; total flap loss occurred in another case.

A minor flap complication occurred in the age group II (7,7%): an haematoma occurred at the recipient site and was evacuated at the patient bed, with no surgical re-exploration.

One patient, in the age group II, had a systemic complication that resulted in respiratory failure soon after the transfer to the intensive care unit. This patient with COPD was an heavy smoker.

Speech, swallowing and chewing assessed in our patients 6 months after surgery resulted well preserved.

Head and neck tumours are often diagnosed late, because of their lack of symptoms in the early stage. In these cases a large demolition is needed. Because of the importance of the quality of life, surgery has to be safe and give satisfactory functional outcomes. Microvascular free-tissue transfer has gained a central role for these large reconstructions to protect important functions of this region. The success rate of this surgery at the present time is reported to be in the range of 91% to 99% in large series from major microsurgical centers [3-5].

Age has been regarded frequently as an independent risk factor for bad surgical outcomes; before the 1960s the mortality rate for elderly patients undergoing elective surgery was 2-6 times higher than in the general population [6]. The reasons that may explain this difference are well documented; the most common medical problems that affect the mortality are heart failure, or the compromise of pulmonary function [7,8].

The improvements in anaesthesia techniques explain the reduction of the mortality rate in elderly patients during these 40 years, especially for patients with cardiac disease [9].

Studies on microsurgical free flap conducted in elderly patients do not agree in defining the term "elderly", but they demonstrated that age is not an important factor influencing the success of this surgery [10-13]. Pompei et al. in a large study of pedicled and free flaps for head and neck reconstructions showed that complications were related with comorbidities more than the age [14].

A reliable predictor of postoperative morbidity could be the ASA status as suggested in a study by Serletti et al. [13].

However, these studies stress how several factors can lead to free-flap complication; most of them reported that age does not impact the free transfer success, but results are variable.

Our results are comparable to the ones showed by Shestak et al. in their small series of cases. In this study 19 patients underwent a free flap reconstruction and results showed a 16% major surgical complication rate in patients over 70 years and 13% in patients under 70 years [10].

In the present study one patient died after this surgery (mortality rate 3,5%); this fatal post-operative complication was correlated with a higher ASA class.

## Conclusion

In conclusion our study shows that microsurgical free-flap transfer can be considered a safe technique for head and neck reconstruction in all age groups. Pre-existing systemic disease could influence peri- and post-operative complications, and ASA status could be a well accepted way to select patients undergoing this surgery.

**Figure 1:**
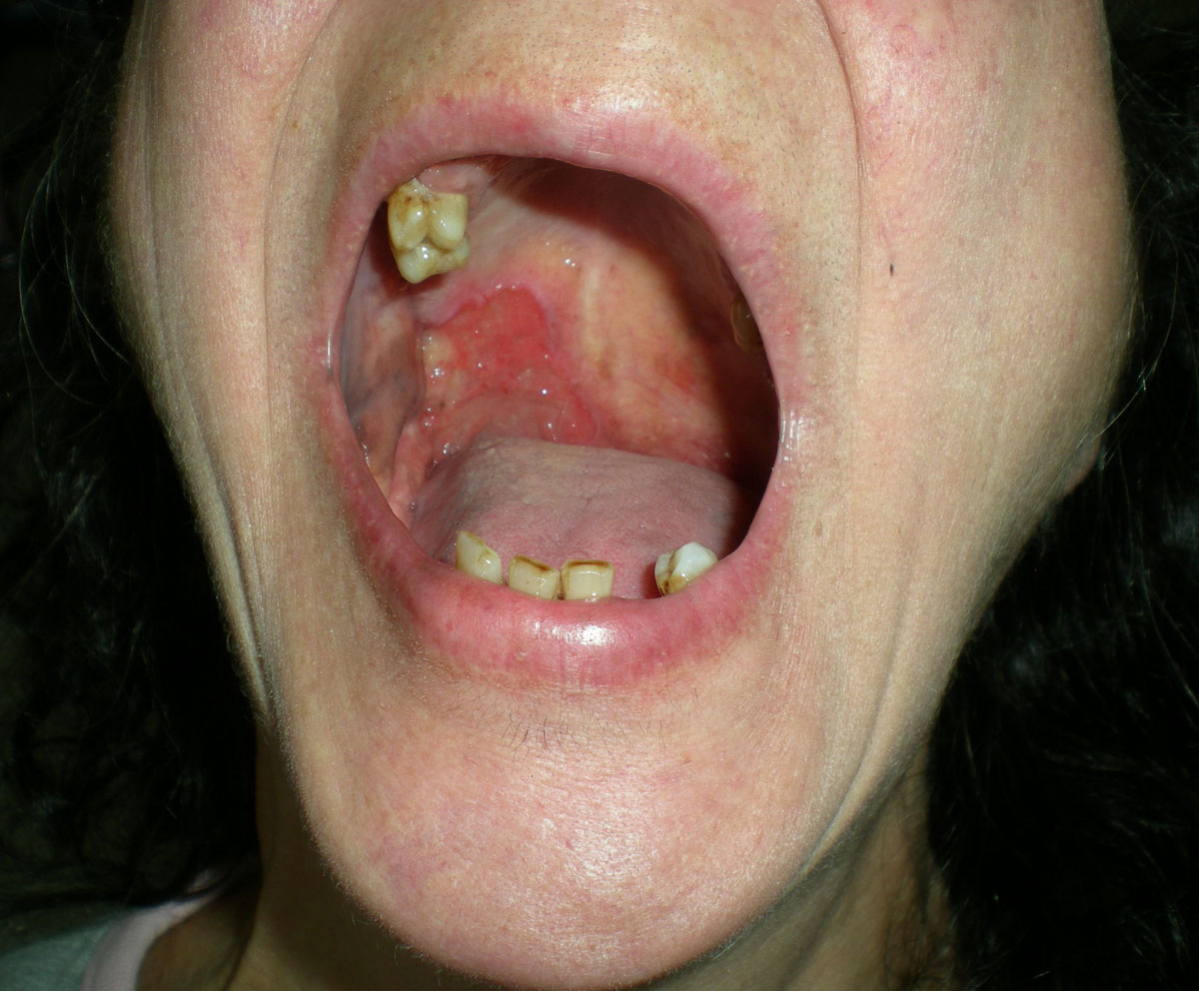
**Squamous cell carcinoma of the right cheek and tonsillar pillar**.

**Figure 2:**
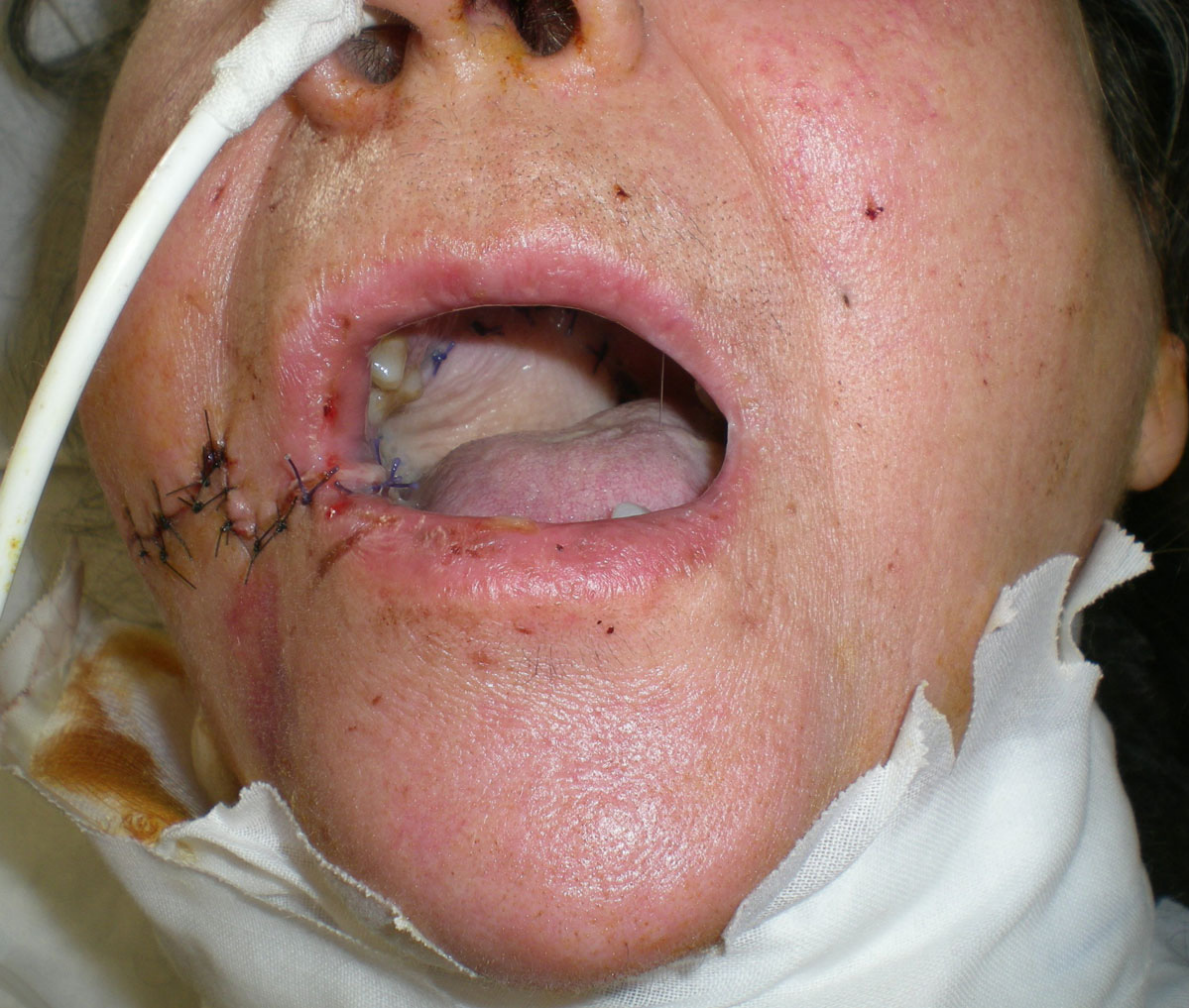
**Transferred radial forearm free flap at two weeks**.

**Figure 3:**
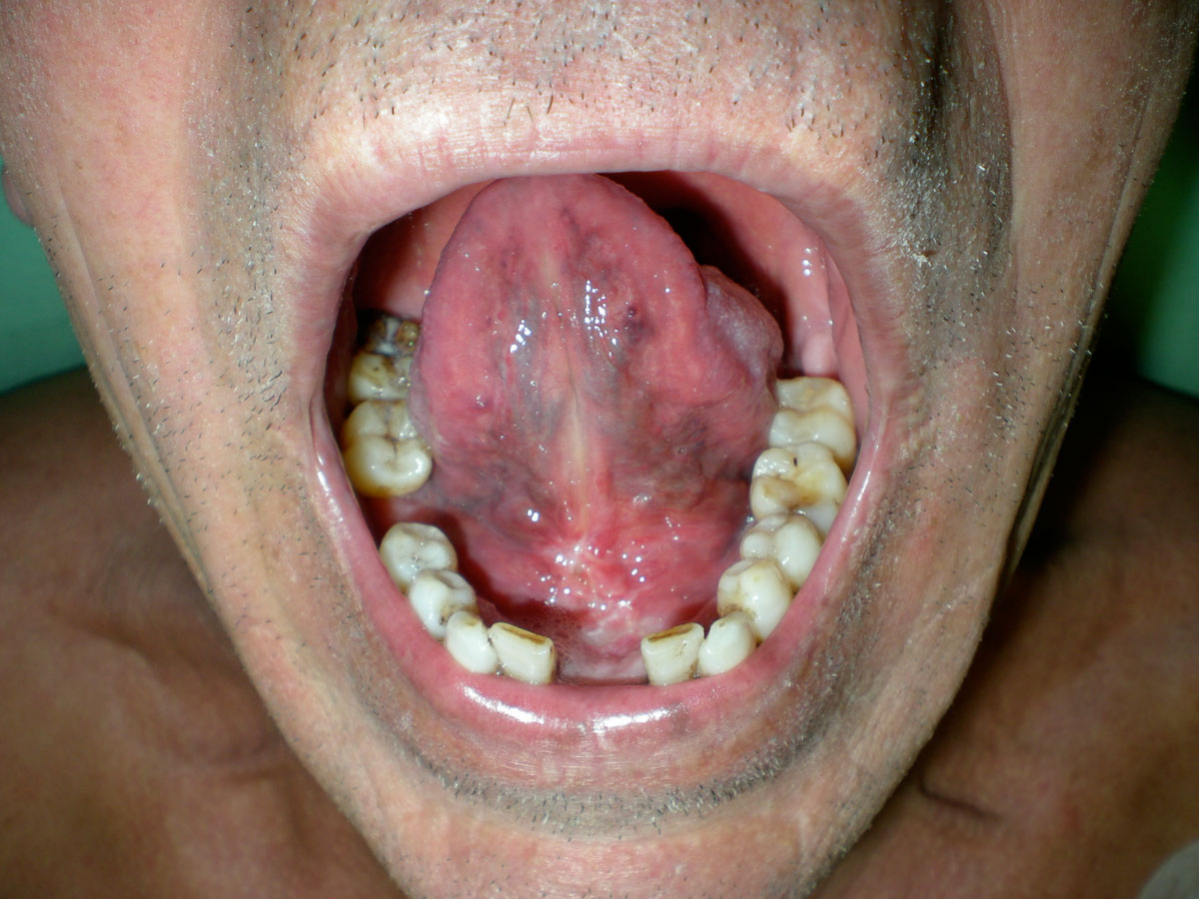
**Squamous cell carcinoma of the floor of the mouth**.

**Figure 4:**
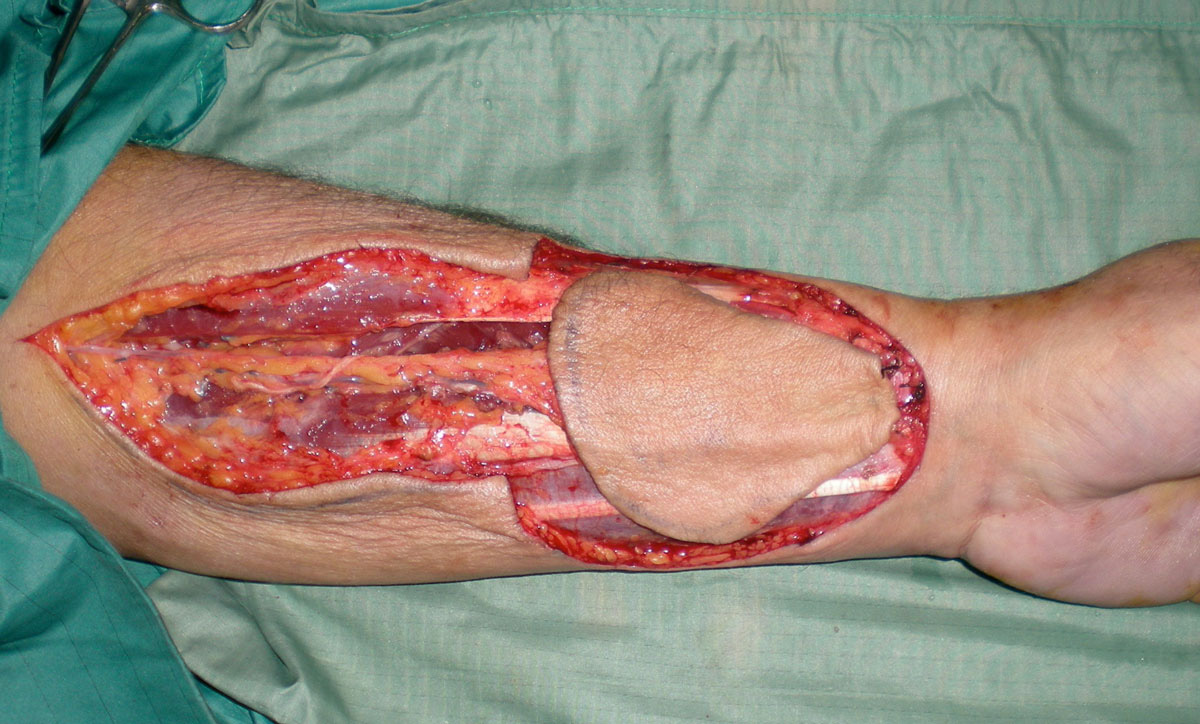
**Radial forearm free flap harvesting and its pedicle**.

**Figure 5:**
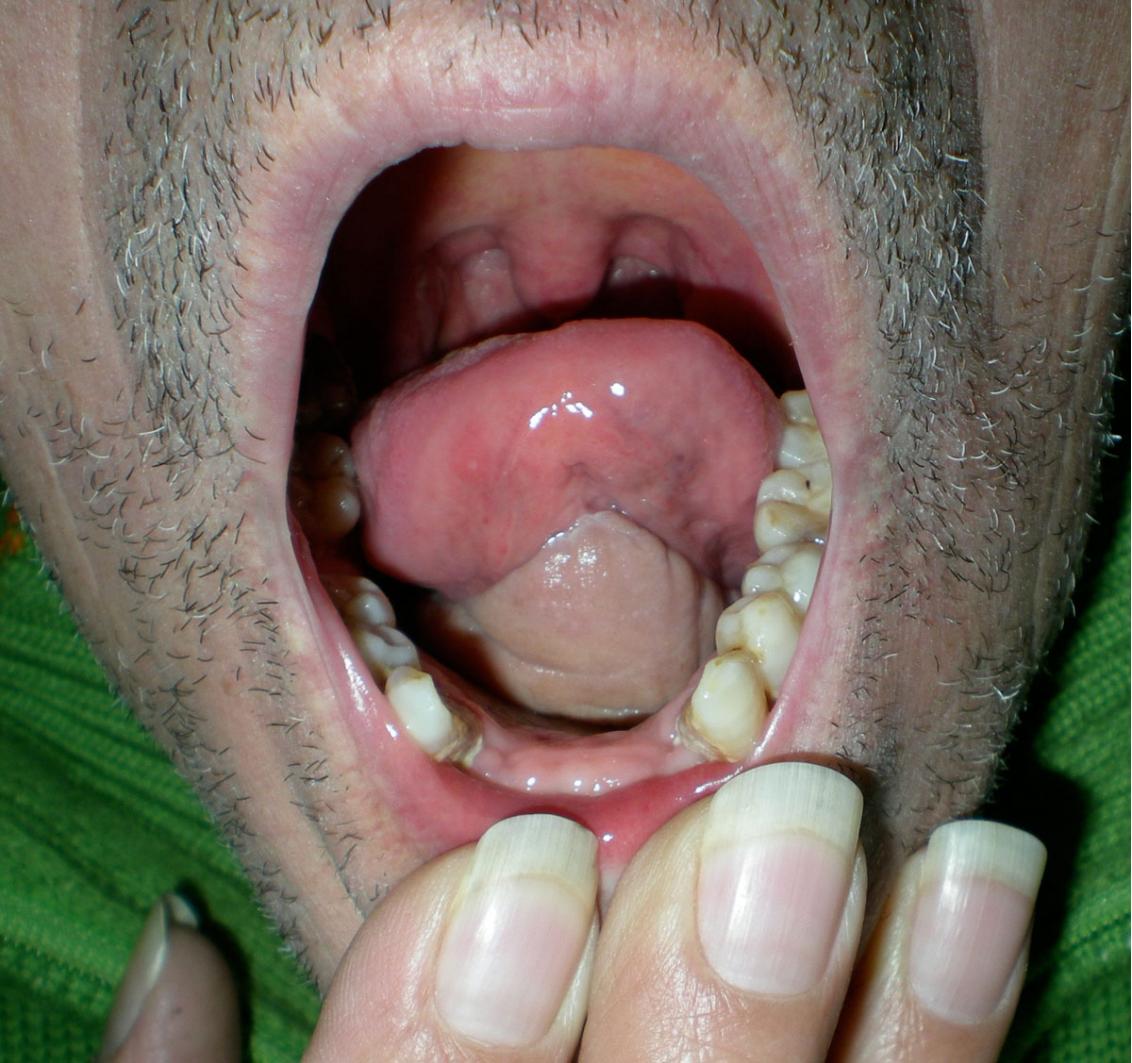
**Settled radial forearm free flap at 6 months follow-up**.

**Figure 6:**
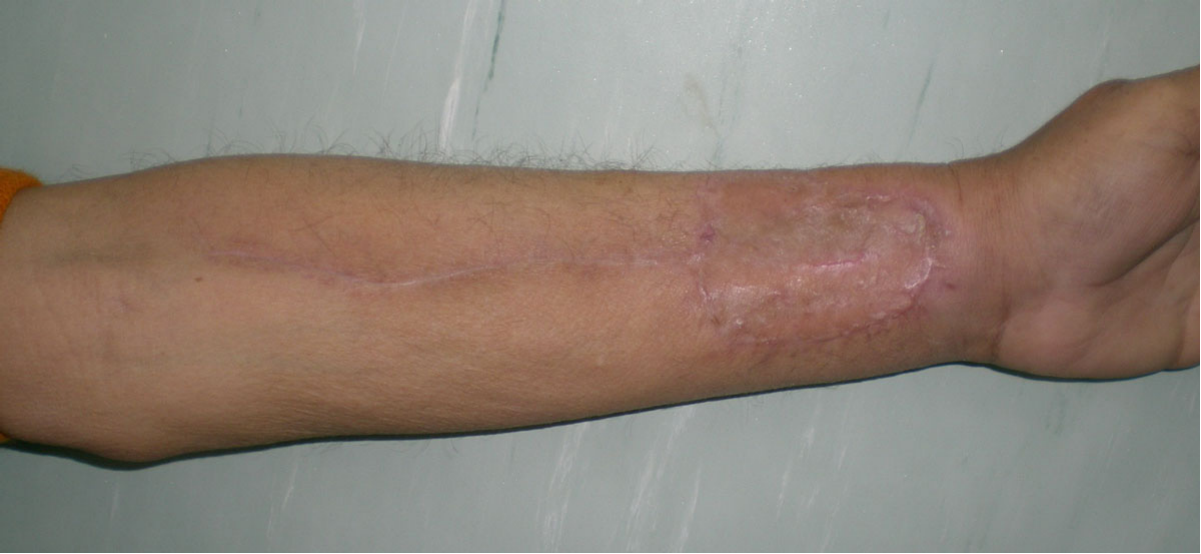
**Healed skin grafted radial forearm free flap donor site 6 months after surgery**.

## List of abbreviations used

ASA= American Society of Anesthesiologists; COPD= Chronic Obstructive Pulmonary Disease; SCC= Squamous Cell Carcinoma; BCC= Basal Cell Carcinoma.

## Competing interests

The authors declare that they have no competing interests.

## Authors' contributions

F.T.: conception and design, acquisition and interpretation of data, drafting the manuscript, given final approval of the version to be published. S.L.P.: acquisition of data, given final approval of the version to be published. S.R.: interpretation of data, drafting the manuscript, given final approval of the version to be published. P.B, S.M, L.C, P.G: critical revision, interpretation of data, given final approval of the version to be published. G.N.: drafting the manuscript, given final approval of the version to be published. G.D.A.O.: acquisition and interpretation of data, critical revision, given final approval of the version to be published. F.S.: conception and design, acquisition and interpretation of data, drafting the manuscript, critical revision, given final approval of the version to be published.

## Authors' information

FT: Medical Doctor. SLP: Resident in Plastic, Reconstructive and Aesthetic Surgery at University Federico II, Naples. SR: Resident in Plastic, Reconstructive and Aesthetic Surgery at University Federico II, Naples. PB: Resident in Maxillofacial Surgery at University Federico II, Naples. GN: Medical Student. SM: Resident in Plastic, Reconstructive and Aesthetic Surgery at University Federico II, Naples. LC: Specialist in Plastic, Reconstructive and Aesthetic Surgery at University Federico II, Naples. PG: Specialist in Maxillofacial Surgery at University Federico II, Naples. GDAO: Assistant Professor in Maxillofacial Surgery at University Federico II, Naples. FS: Assistant Professor in Plastic, Reconstructive and Aesthetic Surgery at University Federico II, Naples

## Declarations

Publication of this article was funded by research university funding.

This article has been published as part of *BMC Surgery *Volume 13 Supplement 2, 2013: Proceedings from the 26th National Congress of the Italian Society of Geriatric Surgery. The full contents of the supplement are available online at http://www.biomedcentral.com/bmcsurg/supplements/13/S2.
